# Read by QxMD

**DOI:** 10.5195/jmla.2020.930

**Published:** 2020-04-01

**Authors:** Carolyn A. Klatt

**Affiliations:** Associate Professor, Reference; Electronic Resources Librarian; and Associate Director, Savannah Campus, Skelton Medical Libraries, Mercer University School of Medicine, Savannah, GA, klatt_ca@mercer.edu, http://orcid.org/0000-0003-0015-2103

## INTRODUCTION

Launched in November 2012, Read by QxMD is a free app and web service that operates like a personal awareness aid, providing a single place for health care providers to keep up with new medical and scientific research, read topic reviews, search PubMed, and add keywords to follow. Articles may be saved in portable document format (PDF) to read offline, annotate, highlight, or share with colleagues via email, Twitter, and Facebook. After registration is completed, an algorithm personalizes a feed of recommended articles based on the information provided. The more information that is provided, the more tailored the recommendations on the feed will be.

Initially, access to free open access papers is provided. An institution can subscribe for free to the Basic Institutional Edition. Users affiliated with that institution can then access journals subscribed to by that institution and share ideas and feedback in the comments section. Articles can be saved and placed in a “Personal Collection,” which can be made publicly shareable on the web. A user does not need Read to access a list of citations in a particular collection. Read is an approved educational activity, so physicians based in the United States can receive up to twenty hours of continuing medical education credits [1]. There is also a version that streamlines access to journals subscribed to by an institution and provides analytics and promoted research [2].

## MAJOR FEATURES

**Figure 1:**
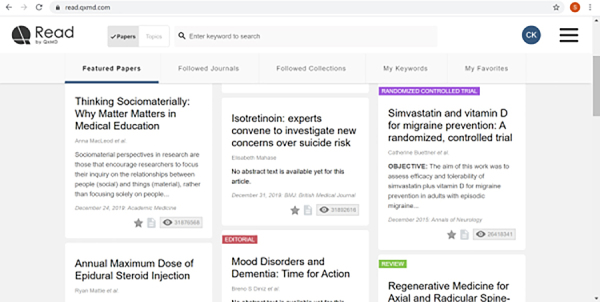
Read by QxMD web interface

Figure 1 shows the QxMD web interface.

### Featured feed

When a user registers for Read, the app personalizes a feed of recommended articles based on information provided such as profession, specialization, and additional areas of interest. The content is determined by Read’s proprietary algorithm. The Featured Feed will include the most popular papers in various categories, such as best papers of all time, best papers monthly from a specialty, and trending papers in a specialty. Most of these papers will be those with the highest interactions and views in the profession and specialty the user indicated at sign up. As the user has more interactions, the recommendations in the feed will become more personalized, based on actions such as reading a paper, giving a paper a thumbs up, sharing an article, or adding an article to a collection [3].

### Followed journals

The Followed Journals feature acts as a journal current awareness service. When a journal is selected, the user is notified as new content is added. Journal preferences can be edited at any time.

### Followed collections

Users can choose from thousands of curated selections of articles created by members of the QxMD community or curate their own personal collection of articles and share it with peers or other users in Read. For each collection, users can view authors, number of papers, and how many followers each collection has. Papers are available from the PubMed database and can be instantly added to a collection. Read automatically syncs with other applications that make links to collections shareable, even if the recipient does not have Read. Push Notifications can be set so that followers of a collection will be notified of updates. Only article citations are shared, so collections are copyright compliant. When a paper in a collection is read, Read then recommends articles that are similar to the paper in the collection [4].

### Search and My Keywords

Users can search articles from PubMed and Read’s database of topic reviews by entering simple terms or building more complex queries using Boolean operators, an asterisk as a truncation symbol at the end of a word to include word stems, parentheses, phrase searching with quotes, or combining all of the above. By using the My Keywords features, users are notified when new articles that match the keywords are available. Read also uses keywords to further enhance recommendations in the Featured Feed [5].

### My Favorites

Articles can be saved for later review by clicking on the star icon on the article. The article can be added to a collection, or a new collection can be created. With the Android or iOS app, users can save articles for reading offline and can draw, underline, highlight, or add notes to PDFs of available full-text papers. A collection can be shared or made public, so that other users can see it [6]. Users can join a discussion of the article, post comments, and see what others are saying about the article.

## CONTINUING MEDICAL EDUCATION

Individuals can receive up to twenty hours of CME or American Medical Association (AMA) Physician’s Recognition Award (PRA) category 1 credits by reading articles through Read. This is only available through the mobile versions of Read on Android and iOS. To receive credits, the user must be a physician based in the United States, have CME Tracking turned on in the Read app, and submit CME credits by going to readbyqxmd.com/cme [7].

## PRIVACY

**Figure 2:**
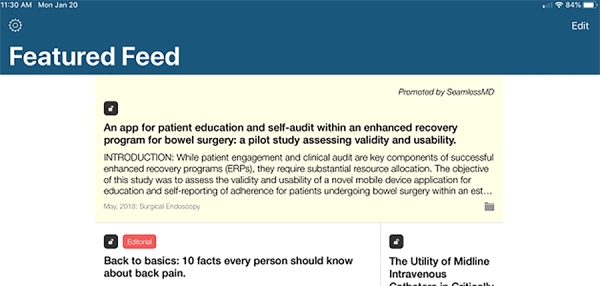
Read by QxMD sponsored content iOS interface

A user’s registration information is stored by QxMD primarily to provide personalized content delivery. However, a secondary use of the information is to allow QxMD to target a user with specific advertising. When users engage with content on the QxMD platform, they may be “presented with advertisements, promoted research and/or opportunities to engage in industry-sponsored informational programs consisting of sponsor-selected materials” [8]. QxMD also reserves the right to change its privacy policy at any time [8]. While companies can highlight articles to raise awareness, it is up to the user to choose to engage. Since Read allows groups to promote specific articles, an organization or company, even drug companies could promote articles. The promoted material will, however, be peer reviewed [9]. There is a yellow tinged highlight and a disclosure message at the top right for sponsored content (Figure 2).

## INSTITUTIONAL EDITION

An Institutional Edition with three levels is available for libraries, hospitals, and health care organizations. The free Basic Level provides one administrator account and allows an institution to create a campaign to promote research and improve awareness; integrate with any proxy, virtual private network (VPN), or single sign-on (SSO)/Shibboleth; index PubMed holdings; integrate IPs; access analytics; obtain user and librarian support; and retrieve community breakdown data and usage data. Users can request pricing for upgrades to Gold or Platinum. The Gold level provides two administrator accounts, four promoted research campaigns, interlibrary loan integration, and all the other features of the Basic level, plus journal performance, vendor insights, user behaviors, and custom filtering of accession data. The Platinum level provides unlimited administrator accounts, unlimited promoted research campaigns, and all the other features of the Gold level, plus custom integrations [10].

## CONCLUSIONS

The amount of information available to health care professionals is immense, with the PubMed database alone containing more than thirty million citations and abstracts of peer-reviewed biomedical literature [11]. Sifting through the literature to find pertinent information for patient care and research is challenging. In an age of information overload, Read provides help for busy health care professionals to access personalized journal articles in one location. Articles have labels such as Review, Editorial, and Randomized Controlled Trial. Read makes use of social media, allowing users to post comments about articles and see what others are saying.

In this reviewer’s opinion, an Institutional Edition is a must because it allows automatic access to full-text articles subscribed to by the institution, a visual indicator to see which papers are available through institutional holdings, and support. The analytics provide information about which journals are read the most and the least, as well as journals that readers would like to access but are unable to.

Read is designed for health professionals on the go, so the Android or iOS mobile versions should be used to take full advantage of all features, such as easy annotation of articles and CME.

In August of 2019, WebMD Health Corporation, an Internet Brands company, acquired QxMD. Terms of the transaction were not made public, but available information indicates that QxMD will continue to operate as an independent subsidiary of WebMD [12]. WebMD’s Medscape provides news and perspectives by specialty, point-of-care drug and disease information, and relevant professional education and CME [13], so it will be interesting to see what might develop from this acquisition.

## 

***Carolyn A. Klatt, MLIS****, klatt_ca@mercer.edu, http://orcid.org/0000-0003-0015-2103, Associate Professor, Reference; Electronic Resources Librarian; and Associate Director, Savannah Campus, Skelton Medical Libraries, Mercer University School of Medicine, Savannah, GA*
